# Treatment with Aortic Stent Graft Placement for Stanford B-Type Aortic Dissection in a Patient with an Aberrant Right Subclavian Artery

**DOI:** 10.1155/2015/746354

**Published:** 2015-10-19

**Authors:** Yohei Kawatani, Yujiro Hayashi, Yujiro Ito, Hirotsugu Kurobe, Yoshitsugu Nakamura, Yuji Suda, Takaki Hori

**Affiliations:** Department of Cardiovascular Surgery, Chiba-Nishi General Hospital, 107-1 Kanegasaku, Matsudo-Shi, Chiba-ken 2702251, Japan

## Abstract

A 71-year-old man visited our hospital with the chief complaint of back pain and was diagnosed with acute aortic dissection (Debakey type III, Stanford type B). He was found to have a variant branching pattern in which the right subclavian artery was the fourth branch of the aorta. We performed conservative management for uncomplicated Stanford type B aortic dissection, and the patient was discharged. An ulcer-like projection (ULP) was discovered during outpatient follow-up. Complicated type B aortic dissection was suspected, and we performed thoracic endovascular aortic repair (TEVAR). The aim of operative treatment was ULP closure; thus we placed two stent grafts in the descending aorta from the distal portion of the right subclavian artery. The patient was released without complications on postoperative day 5. Deliberate sizing and examination of placement location were necessary when placing the stent graft, but operative techniques allowed the procedure to be safely completed.

## 1. Introduction

Acute aortic dissection, in which the tunica intima and tunica media are separated due to a tear in the tunica intima, is characterized by abdominal or chest pain and high mortality [1]. Conservative management is recommended in patients with Stanford type B dissections, where dissection of the ascending aorta is not present. Operative treatment is recommended for complicated cases in which findings such as reperfusion injury, continuing chest pain, or a progression of dissection are present and the risk of rupture is high [2].

There are many subtypes of aortic arch branching patterns. Here, we describe a patient who had an aberrant right subclavian artery (ARSCA) and developed acute aortic dissection. Placement of stent grafts in the aorta after emergence of an ulcer-like projection (ULP) during the subacute phase was an effective treatment. Concomitant ARSCA and acute dissection are rare. Patients with this condition who receive a stent graft are even more rare.

## 2. Case Presentation

A 71-year-old hypertensive man presented to hospital with a 1-day history of chest pain and dyspnea. Enhanced computed tomography (CT) revealed type B aortic dissection. He was transferred to our hospital for further investigation and care. His medical history included medically managed hypertension, untreated diabetes mellitus, and a 50-year smoking habit (Brinkman index: 1500).

**Figure 1:**
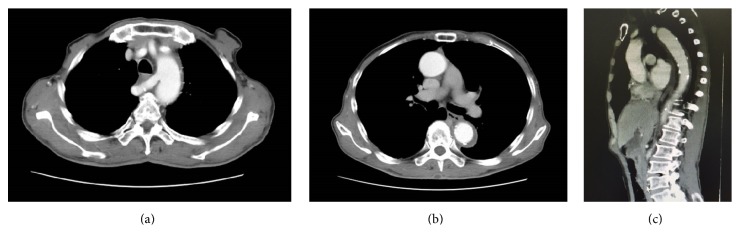
Contrast-enhanced CT images at time of onset. (a, b) Horizontal cross section. The origin of the right subclavian artery is identified as the fourth branch of the aortic arch and runs posteriorly to the right of the esophagus. No dissection of the right subclavian artery was present. Thrombosis of the false lumen from the origin to periphery of the right subclavian artery was observed. (c) Sagittal section. False lumen was observed. Total false lumen thrombosis was observed, and no signs of ULP were noted.

On presentation, blood pressure was controlled below 120 mmHg with nicardipine. The patient complained of back pain. Abdominal and peripheral pulse examinations revealed no abnormality. Enhanced CT showed dissection and coagulation in the false lumen from distal to left subclavian artery at the renal artery level. Contrast-enhanced CT and echocardiographic findings did not reveal any congenital heart defects such as right-sided aortic arch or cardiac malformation. Two arterial anomalies were observed. The right subclavian artery arose from the aortic arch posterolateral to the origin of the left subclavian artery. The right vertebral artery arose from the right common carotid artery (Figure 1). All aortic branches including the aberrant right subclavian artery arose from the true lumen.

Upon admission, antihypertensive and analgesic treatment with bed rest were initiated. The symptoms were gradually ameliorated; therefore we performed rehabilitation in accordance with the type B acute aortic dissection rehabilitation protocol utilized by our hospital. Contrast-enhanced CT was performed on hospitalization days 2, 4, 7, and 12 to assess the aortic condition. No changes such as dilation or emergence of ULP were noted. After the rehabilitation protocol was completed, the patient was discharged. Hypotensive drugs were prescribed to control blood pressure, and the patient was scheduled for outpatient follow-up aortic imaging.

**Figure 2:**
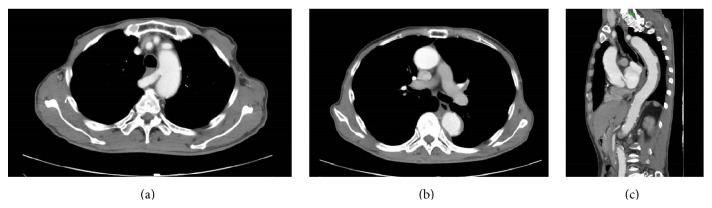
Contrast-enhanced CT findings after onset. (a) Horizontal cross section. False lumen thrombosis was observed. No changes in the origin of the right subclavian artery were noted. (b) Horizontal cross section. (c) Coronal cross section. The emergence of ULP in the descending aorta was observed.

On day 11 after discharge (day 25 after onset), the patient visited our hospital for outpatient follow-up. Contrast-enhanced CT findings revealed a ULP (Figure 2). We determined that the patient had complicated type B dissection, and an urgent operation was required. After the necessary presurgical checkups, we performed endovascular repair under general anesthesia, with tracheal intubation for mechanical ventilation. The right brachial artery was accessed, and a pigtail catheter was positioned in the ascending aorta for contrast enhancement. The left common femoral artery was exposed and used as the access route for stent graft placement.

Contrast-enhanced CT imaging of the thoracic aorta was used to visualize the ULP in the descending aorta. We confirmed the origin of the right subclavian artery that had arisen as the fourth aortic branch. The diameter of the proximal landing zone was 30 mm and the diameter around the ULP was 27 mm. The treatment length was 150 mm, which was between the position of just distal to ARSCA and 50 mm distal to the ULP. A guide wire (Amplatz Super Stiff wire) was inserted from the left common femoral artery to the ascending aorta and used as a guide for the stent grafts (Relay plus 26*∗*26*∗*10, Bolton) that were placed to cover the ULP. Care was taken to place the bare stent in a sufficiently peripheral site to prevent the tip from contacting the aortic arch. Furthermore, the stent was positioned 2 cm medially to the medial part of the ULP.

**Figure 3:**
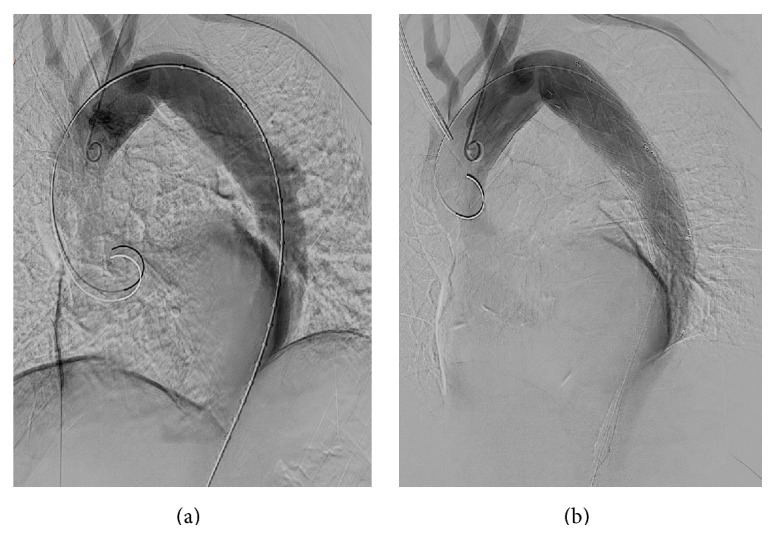
Image of angiographic findings taken during operative treatment. (a) Angiographic findings before treatment. The origin of the right subclavian artery was confirmed, and ULP was observed on contrast-enhanced CT. (b) Angiographic contrast-enhanced CT findings after placement of the stent grafts. The stent grafts were inserted so that the tips did not come into contact with the curve of the arch. The disappearance of the ULP was confirmed by contrast-enhanced CT.

For Proximal part, we selected a stent graft that was considered appropriate for long-term treatment, at the aortic diameter (Valiant 32*∗*32*∗*10, Medtronic). We placed the second stent graft in the descending aorta from the region distal to the origin of the ARSCA. Angiography using a pigtail catheter confirmed that the ULP had disappeared on contrast-enhanced imaging and that no occlusion existed in the fourth branch of the aortic arch (Figure 3).

**Figure 4:**
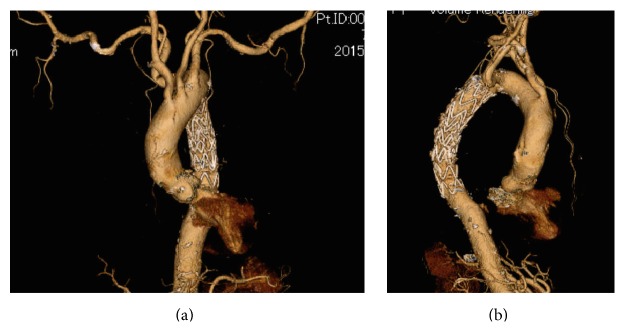
Three-dimensional CT findings at postoperative day 12. (a) Anterior view. (b) Right lateral view. The disappearance of ULP was confirmed by contrast-enhanced CT, and no endoleaks were observed. The stent grafts were placed distal to the origin of the right subclavian artery so the tips of the bare stents did not come into contact with the aortic wall.

No postoperative complications, including cerebral infarcts, paraplegia, or right upper limb claudication, were observed. On hospital day 5, the patient was discharged in ambulatory condition. At postoperative day 12, contrast-enhanced CT findings revealed that the ULP had disappeared, and no other abnormal findings such as occlusion or stenosis of the arch branch were observed (Figure 4).

## 3. Discussion

**Figure 5:**
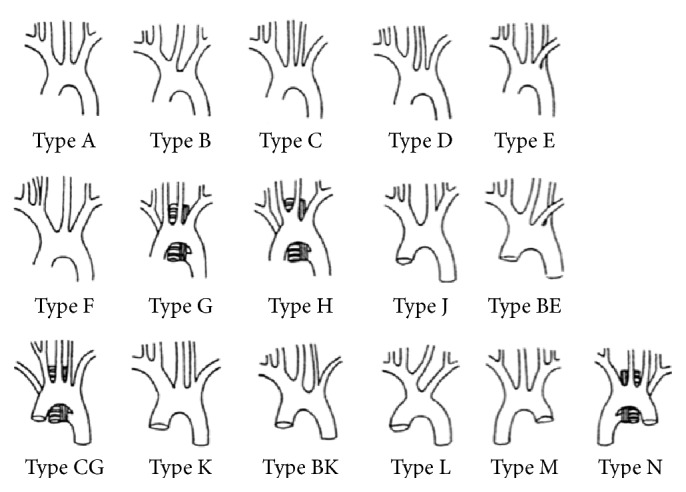
A schematic drawing of aortic arch branch classifications by Adachi, Williams, and Nakagawa. Cited from Williams et al. [5].

The majority of cases of ARSCA are asymptomatic. ARSCA is the most common congenital aortic arch anomaly, found in 0.5–1.8% of humans [3]. ARSCA is due to the persistence of the embryonic right dorsal aorta with involution of the arch segment between the right common carotid artery and right subclavian artery [4]. In the present patient, the right subclavian artery was the fourth branch of the aortic arch, and after branching from the most distal region of the artery arch, it ran posteriorly to the right of the esophagus. The right vertebral artery did not originate from the right subclavian artery, but rather from the right common carotid artery, which was the first branch. There are many variations of the aortic arch branches, which are classified according to the system proposed by Williams et al. (Figure 5) [5]. The present patient was similar to type G or type GC. However, in type G, the right vertebral artery branches from the subclavian artery, whereas, in the present patient, the right vertebral artery originated from the common carotid artery. In type GC, the right vertebral artery branches from the right subclavian artery, similar to the present case, but type GC differs in the fact that the left vertebral artery branches normally from the subclavian artery. While type G is the most common of all the variations, it seems that there are few cases that completely match the type seen in the present patient. The frequency of this morphology has not been elucidated. ARSCA can be involved in aortic dissection, either as the site of the primary intimal tear or as a dissected aortic branch [6, 7].

Conservative treatment is recommended for uncomplicated type B dissection [2]. Since our patient was hospitalized directly after symptom onset, the case was considered to be uncomplicated, and therefore we performed conservative treatment. While operative treatment is recommended for complicated type B dissection, the incidence of complications associated with operative treatment is high, and the indications for operative treatment and surgical methods remain controversial. In recent years, TEVAR has been reported as effective. In our department, operative treatment is generally only performed in patients whose findings reveal the appearance or exacerbation of symptoms such as the emergence of pain, enlargement of ULP, or enlargement of arterial diameter. There is no consensus concerning the intervention period for performing TEVAR.

We administer antihypertensives, order bed rest, and continue conservative management of symptoms because, during the postoperative acute phase (within 2 weeks after onset), the arterial wall that has dissected is inflamed and fragile, even in cases where findings associated with the abovementioned complicated type are present. After the acute phase has passed, we then perform TEVAR. In exceptional cases when the risk of rupture is determined to be high based on the findings, the surgeon may perform TEVAR during the acute phase.

Our patient received conservative treatment and rehabilitation during the 2-week period of hospitalization after the onset of symptoms, and contrast-enhanced CT findings during follow-up were stable. The patient was discharged with only conservative treatment. However, because contrast-enhanced CT images taken during outpatient treatment on day 25 after onset revealed the emergence of ULP, we elected to perform urgent TEVAR.

No consensus has been reached concerning the TEVAR sealing zones in cases of Stanford type B aortic dissection. The policy for clinical practice in our department is to take the prevention of paraplegia into consideration and maintain spinal cord perfusion as much as possible by reducing the number of obstructed lumbar and intercostal arteries, limiting entry to a region as small as possible, and performing coverage of entry closure as much as possible. The sealing zone is specified to be 3 cm or longer, and in cases where the condition of the tunica intima is favorable or space is limited due to the position of the stent graft or the anatomical shape of the aorta, it is possible for the length to be shortened to 2 cm. The smallest stent graft size that can be placed is carefully considered to meet these conditions.

In the present patient, a proximal sealing zone of 3 cm would mean that the tip of the bare stent came into contact with the arterial wall of the distal region of the greater curvature of the aortic arch. For this reason, in order to obtain a sealing zone of 3 cm or longer, it was necessary to place the stent from a position on the top of the aortic arch to the median side. However, to shorten the length of long-term treatment, it was necessary to place the stent in a more distal position so that the tip of the bare stent did not come into contact with the distal wall of the curve.

In order to place a stent in the center, it was necessary to obstruct the right subclavian artery. When performing obstruction, coil embolization was necessary to prevent type II endoleaks. It has been reported that, in this variation, the right subclavian artery puts pressure on the esophagus [8]. Moreover, it has been reported that placement of stent grafts from the right subclavian artery to the aorta in this type of patient has led to esophageal perforation [9]. For this reason, we thought that, by placing a coil in the right subclavian artery, the risk of esophageal perforation could be avoided, and therefore we considered distal placement to be preferable. When placing a stent in the periphery of the distal region of the arch, the expected landing zone was 25 mm, shorter than the desired 30 mm, but the condition of the arterial wall was favorable and sealing seemed to be possible. Indeed, angiographic findings after placement of the stent grafts revealed that the ULP had disappeared, and entry closure could be sufficiently performed.

There is a high incidence of pulmonary, renal, and neural complications and early postoperative mortality associated with the conventional operative treatment of Stanford type B acute aortic dissections through thoracotomy [10]. Furthermore, many studies have reported a high risk of damage to the aberrant artery in patients who present with aberrant branching of the aortic arch. By utilizing TEVAR, there was no large difference in risks associated with operative treatment between patients with ordinary branching and the present patient. Even when guiding the wire, there were no adverse events such as migration of the wire into the right subclavian artery, and the procedure could be safely completed.

This case indicates that TEVAR is an effective treatment method for aortic dissection in patients who have ARSCA. However, the long-term prognoses of patients who have B-type aortic dissection have not been elucidated, and future observations and examination are needed.

## 4. Conclusion

It is the authors' opinion that TEVAR is an effective treatment for Stanford type B (complicated type) acute aortic dissection in patients who have ARSCA. As far as we pay attention to the aberrant anatomy preoperatively and during the operation, we can perform straightforward procedure in a patient with a type B dissection that is complicated and has abnormal rare anatomy. Future observations and examination of long-term prognoses are needed.

## References

[1] Kieffer E., Rutherford R. B. (2000). Dissection of the descending thoracic aorta. *Vascular Surgery*.

[2] Hiratzka L. F., Bakris G. L., Beckman J. A. (2010). ACCF/AHA/AATS/ACR/ASA/SCA/SCAI/SIR/STS/SVM guidelines for the diagnosis and management of patients with Thoracic Aortic Disease: a report of the American College of Cardiology Foundation/American Heart Association Task Force on Practice Guidelines, American Association for Thoracic Surgery, American College of Radiology, American Stroke Association, Society of Cardiovascular Anesthesiologists, Society for Cardiovascular Angiography and Interventions, Society of Interventional Radiology, Society of Thoracic Surgeons, and Society for Vascular Medicine. *Circulation*.

[3] Cronenwett J. L., Johnston K. W., Rutherford R. B. (2010). *Rutherford's Vascular Surgery*.

[4] Farber A., Wagner W. H., Cossman D. V. (2002). Isolated dissection of the abdominal aorta: clinical presentation and therapeutic options. *Journal of Vascular Surgery*.

[5] Williams G. D., Aff H. M., Schmeckebier M., Edmonds H. W., Graul E. G. (1935). Variations in the arrangement of the branches arising from the aortic arch in American whites and negroes. *The Anatomical Record*.

[6] Weinberger G., Randall P. A., Parker F. B., Kieffer S. A. (1977). Involvement of an aberrant right subclavian artery in dissection of the thoracic aorta. *American Journal of Roentgenology*.

[7] Kawamoto S., Bluemke D. A., Fishman E. K. (1998). Aortic dissection involving an aberrant right subclavian artery. CT and MR findings. *Journal of Computer Assisted Tomography*.

[8] Donnelly L. F., Fleck R. J., Pacharn P., Ziegler M. A., Fricke B. L., Cotton R. T. (2002). Aberrant subclavian arteries: cross-sectional imaging findings in infants and children referred for evaluation of extrinsic airway compression. *American Journal of Roentgenology*.

[9] Morisaki A., Hirai H., Sasaki Y., Hige K., Bito Y., Suehiro S. (2014). Aortoesophageal fistula after endovascular repair for aberrant right subclavian artery aneurysm. *Annals of Thoracic and Cardiovascular Surgery*.

[10] Won J. Y., Lee D. Y., Shim W. H. (2001). Elective endovascular treatment of descending thoracic aortic aneurysms and chronic dissections with stent-grafts. *Journal of Vascular and Interventional Radiology*.

